# Ovarian cancer risk in Polish *BRCA1 *mutation carriers is not associated with the prohibitin 3' untranslated region polymorphism

**DOI:** 10.1186/1471-2407-8-90

**Published:** 2008-04-08

**Authors:** Anna Jakubowska, Jacek Gronwald, Janusz Menkiszak, Bohdan Górski, Tomasz Huzarski, Tomasz Byrski, Axel Benner, Jan Lubiński, Rodney J Scott, Ute Hamann

**Affiliations:** 1 Pomeranian Medical University, Department of Genetics and Pathology, ul. Polabska 4, 70-115 Szczecin, Poland; 2 Pomeranian Medical University, Department of Surgical Gynecology and Gynecological Oncology of Adults and Adolescents, ul. Powstancow Wlkp. 72, 70-115 Szczecin, Poland; 3 German Cancer Research Center, Division of Molecular Genome Analysis, Im Neuenheimer Feld 580, 69120 Heidelberg, Germany; 4 German Cancer Research Center, Division of Biostatistics, Im Neuenheimer Feld 280, 69120 Heidelberg, Germany; 5 Discipline of Medical Genetics, School of Biomedical Sciences, University of Newcastle, and the Hunter Medical Research Institute, Lookout Road, New Lambton, 2305 NSW, Australia

## Abstract

**Background:**

The variable penetrance of ovarian cancer in *BRCA1 *mutation carriers suggests that other genetic or environmental factors modify disease risk. The C to T transition in the 3' untranslated region of the prohibitin (*PHB*) gene alters mRNA function and has recently been shown to be associated with hereditary breast cancer risk in Polish women harbouring *BRCA1 *mutations.

**Methods:**

To investigate whether the *PHB *3'UTR polymorphism also modifies hereditary ovarian cancer risk, we performed a case-control study among Polish women carrying one of the three common founder mutations (5382insC, 300 T > G, 4154delA) including 127 ovarian cases and 127 unaffected controls who had both breasts and ovaries intact. Controls were matched to cases by year of birth and *BRCA1 *mutation. Genotyping analysis was performed using PCR-based restriction fragment length polymorphism analysis. Odds ratios (OR) were calculated using conditional and penalized univariable and multivariable logistic regression.

**Results:**

A comparison of the genotype frequencies between cases and controls revealed no association of the *PHB *3'UTR _CT+TT genotypes with ovarian cancer risk (OR_adj _1.34; 95% CI, 0.59–3.11).

**Conclusion:**

Our data suggest that the *PHB *3'UTR polymorphism does not modify ovarian cancer risk in women carrying one of the three Polish *BRCA1 *founder mutations.

## Background

Women harboring *BRCA1 *germ line mutations have a high life time risk of developing ovarian cancer, ranging from 16% in specific ethnic groups [1] to 63% in highly selected families with multiple affected individuals [2]. These differences suggest that the penetrance of *BRCA1 *mutations is modified by other genetic and/or environmental factors. A candidate modifier of hereditary ovarian cancer risk is the prohibitin gene (*PHB*, OMIM 176705). The encoded gene product binds to the retinoblastoma protein, resulting in the suppression of E2F-mediated transcription, and the p53 tumor suppressor, which eventuates in increased p53-mediated transcriptional activity [3-5]. The 3'untranslated region (3'UTR) of the *PHB *gene encodes a tumor suppressive *trans*-acting regulatory RNA molecule [6] and acts as a cell cycle inhibitor between G1 and S phase when microinjected into normal mammary epithelial cells and other cancer cell lines [7,8]. A functional single nucleotide polymorphism (SNP) in the *PHB *gene changing a cytosine to a thymine at position 1630 in the 3'UTR (rs6917) has been identified that lacks the anti-proliferative activity [7] and significantly reduces cell motility [9].

The T allele of the *PHB *3'UTR polymorphism has recently been shown to be associated with an increased risk of breast cancer (OR_adj_, 2.12; 95% CI, 1.23–3.70) [10] in Polish women carrying one of the three common *BRCA1 *founder mutations that account for approximately 90% of all detected *BRCA1 *mutations detected in this country [11,12]. In a North-American study, the T allele was also associated with an increased risk of breast cancer, particularly in women with a breast cancer diagnosis before the age of 50 years who had at least one first-degree relative with the disease [13]. However, these findings were not reproduced in two other case-control studies conducted in Australia and Great Britain [14,15].

Since nothing is known about the influence of the *PHB *3'UTR polymorphism on hereditary ovarian cancer risk, we performed a case-control study among Polish women carrying one of the three common *BRCA1 *founder mutations, comprising 127 ovarian cases and 127 matched controls.

## Methods

### Study participants

The Hereditary Cancer Registry at the Pomeranian Medical University in Szczecin, Poland contains clinical and epidemiological data collected from 1997 and 2002 from 1,940 individuals carrying one of the three common Polish *BRCA1 *founder mutations: 5382insC, 300 T > G and 4154delA.

From the 1,940 registered *BRCA1 *mutation carriers, 254 female Polish carriers constituting 127 case-control pairs for whom DNA samples were available were selected for this study. Controls were matched to cases by year of birth (within 2 years), and *BRCA1 *mutation (5382insC, 300 T > G, 4154delA). They were included as controls if they were unaffected by any type of cancer and had not undergone prophylactic mastectomy, adnexectomy or tubal ligation prior to the age at which ovarian cancer was diagnosed in the corresponding case. They were considered as cases if they were diagnosed with invasive primary ovarian cancer (excluding borderline ovarian carcinoma) and had not undergone prophylactic mastectomy, adnexectomy or tubal ligation prior to the age of ovarian cancer diagnosis.

The research was approved by the Ethics Committee of Pomeranian Medical University in Szczecin, Poland and all participants gave informed consent prior to enrolling in the study. All women received genetic counseling prior to and at the provision of their test results.

### Genotyping analysis

Genotyping of the 1630_C > T polymorphism (rs6917) in the 3'UTR of the *PHB *gene (Genbank accession number NT_010783.13) in 254 *BRCA1 *mutation carriers was performed by PCR-based restriction fragment length polymorphism analysis as previously described [10].

Genotyping was carried out by two independent personnel who were blinded to the disease status of the samples. Several samples were sequenced on an ABI prism 377 DNA Sequencer (Perkin-Elmer, Foster City, USA) to confirm the genotypes. The reproducibility of the genotyping data was assessed by repeated analysis of 40/254 (16%) randomly selected DNA samples. Concordance for these quality control samples was 100%. Genotypes were obtained for all 254 *BRCA1 *mutation carriers constituting PCR concordance rate of 100%.

### Statistical analysis

Statistical modelling of the study on 127 ovarian cancer case-control pairs revealed a 55.3% power to detect an OR of 2.00 and an 81.5% power to detect an OR of 2.50. This is based on the assumption that the probability of observing CT+TT genotypes among control patients is 0.2. For the correlation, phi, between matched case and control patients to observe these genotypes we assumed a value of phi = 0.2 according the recommendation of Dupont [16]. This power calculation further assumes the use of a Chi-Square test with a 0.05 significance level.

Risk estimates for the development of ovarian cancer were calculated as odds ratios (OR_crude_) with 95% confidence interval (CI) using a conditional logistic regression model by maximising the conditional likelihood for all 127 case-control pairs. For 83 pairs, we adjusted the risks for potential ovarian cancer risk factors, referred to as adjusted odds ratios (OR_adj_), by including age at menarche, age at first live birth, parity (0, 1, 2, 3, 4, 4+ children), lifetime cumulative months of breastfeeding (≤12 and >12 months), oral contraceptive (OC) use (<5 and ≥5 years), hormone replacement therapy (HRT) use (never, ever), smoking (<4 and ≥4 pack-years) and body mass index (BMI) (at age of breast cancer diagnosis for cases and at corresponding age for controls) in the penalized-likelihood logistic regression model [17].

Age comparisons among carriers of different genotypes were performed using the Mann-Whitney U-test. Exact 95% confidence intervals were calculated for binomial probabilities. The statistical analyses were done using the software package R, version 2.3.1 [18] with packages "survival", version 2.26, and "logistf", version 1.06. In order to account for a potential bias due to the presence of relatives, we performed a second analysis using only unrelated study subjects. After exclusion of the pairs with relatives, OR_crude _were calculated for 104 pairs and OR_adj _for 69 pairs. Two-sided *p *values of 0.05 or less were considered as statistically significant.

## Results

### Description of the study participants

A total of 254 *BRCA1 *mutation carriers including 127 ovarian cancer cases and 127 unaffected controls were included in this study. All mutation carriers were selected from families with at least one ovarian cancer diagnosed at any age or with multiple cases of breast and ovarian cancer. Controls were matched to cases by year of birth and *BRCA1 *mutation and were similar to cases with respect to potential ovarian cancer risk factors including year of birth, age at first live birth, age at menarche, BMI, parity, breastfeeding, and smoking. In contrast, OC use of 5 years and more and HRT use were less frequently reported by ovarian cancer cases than by controls (0 cases, 8 controls; 2 cases and 11 controls, respectively; Table 1). These differences were not considered relevant because of the small number of individuals.

**Table 1:** Comparison of ovarian cancer cases and matched controls

Characteristic	Cases (n = 83) n (%)	Controls (n = 83) n (%)
Year of birth (median)	1953 (range 1928–1971)	1953 (range 1929–1971)
Age of 1^st ^live birth (median)	22 (range 16–38)	23 (range 17–35)
Age at menarche (median)	14 (range 10–17)	14 (range 9–18)
BMI (median)	25 (range 19–38)	25 (range 17–44)
Age (median)	45^a ^(range 25–71)	49^b ^(range 31–73)
Parity		
0	7 (8)	7 (8)
1	11 (13)	12 (14)
2	43 (52)	41 (50)
3	13 (16)	17 (20)
4	7 (8)	4 (5)
>4	2 (3)	2 (3)
Breastfeeding^c^		
≤ 12 months	20 (26)	23 (30)
>12 months	56 (74)	53 (70)
OC use		
<5 years	83 (100)	75 (90)
≥ 5 years	0 (0)	8 (10)
HRT		
Never	81 (97)	72 (87)
Ever	2 (3)	11 (13)
Smoking^d^		
<4 pack-years	56 (67)	52 (63)
≥ 4 pack-years	24 (29)	31 (37)
*BRCA1 *mutation		
5382insC	61 (74)	61 (74)
300T>G	16 (19)	16 (19)
4154delA	6 (7)	6 (7)

BMI: body mass index; OC: oral contraceptive; HRT: hormone replacement therapy.

^a ^Age at diagnosis of ovarian cancer.

^b ^Age at the time of matching.

^c ^Nulliparous women were excluded.

^d ^Missing information on smoking status from three cases.

### Comparison of *PHB *3'UTR T allele and genotype frequencies among ovarian cancer cases and controls

Among *BRCA1 *carriers, no difference in the T allele frequency between ovarian cancer cases and controls was observed (27/254, 11%; 95% CI 7.12–15.10 vs. 25/254, 10%, 95% CI 6.50–14.20) (Fisher's exact test, *p *= 0.88). The genotype distribution among all case-control pairs (n = 127) and among those with risk factor information (n = 83) are shown in Table 2. There was no difference in CT+TT genotype frequencies among cases and controls (OR_crude_, 1.05; 95% CI, 0.54–2.04; OR_adj_, 1.34; 95% CI 0.59–3.11). After exclusion of related study subjects, similar results were obtained (OR_crude_, 1.06; 95% CI 0.55–2.01; OR_adj_, 1.38; 95% CI 0.57–3.41).

**Table 2:** Association of the *PHB*_1630_C > T polymorphism with ovarian cancer risk in *BRCA1 *carriers

	All cases and controls			
	
Genotype	Cases (n = 127) n (%)	Controls (n = 127) n (%)	OR_crude_^b ^(95% CI)	*p*
*PHB*_1630_C > T				
CC	101 (80)	102 (80)	1.00 (reference)	
CT+TT^a^	26 (20)	25 (20)	1.05 (0.54–2.04)	1.00

	Cases and controls with risk factor information			
	
Genotype	Cases (n = 83) n (%)	Controls (n = 83) n (%)	OR_adj_^c ^(95% CI)	*p*

*PHB*_1630_C > T				
CC	64 (77)	65 (78)	1.00(reference)	
CT+TT^a^	19 (23)	18 (18)	1.34 (0.59–3.11)	0.49

^a ^Due to the low proportion of <3% in patients and controls, CT and TT genotypes were combined.

^b ^OR_crude_: crude odds ratio.

^c ^OR_adj_: odds ratio adjusted for age at menarche, age of first live birth, parity, breastfeeding, OC use, HRT, smoking and BMI.

Among all cases, women with the CT+TT genotypes were diagnosed at a median age one year younger than women with the CC genotype [45 years (range 35–71 years) vs. 46 years (range 25–71 years)], but this age difference was not statistically significant (Mann-Whitney test, *p *= 0.77).

## Discussion

In the present study we investigated the effect of *PHB *3'UTR polymorphism on the risk of ovarian cancer in *BRCA1 *mutation carriers. While one investigation of the *PHB *polymorphism in an ovarian cancer population has been previously reported [19], nothing is known about its' influence in women with predisposing mutations in the *BRCA1 *gene.

In this matched case-control study we did not find an association of the *PHB *CT+TT genotypes with ovarian cancer risk in selected women harbouring one of the pathogenic *BRCA1 *founder mutations. This result remained after adjusting for known and putative ovarian cancer risk factors including age at menarche, age of first live birth, parity, breastfeeding, OC use, HRT, smoking status and BMI [20,21] as well as after exclusion of those study participants who were related to one another suggesting that confounding due to these variables was unlikely.

Outside the context of hereditary ovarian cancer there is only one Australian study that has investigated the contribution of the *PHB *3'UTR polymorphism to ovarian cancer risk. In this case-control study on 553 ovarian cancer cases and 300 unaffected controls also no association of the CT+TT genotypes with ovarian cancer risk has been found [19]. When taken together, it appears that our results and those reported by Spurdle et al. [19] do not support a role for *PHB *polymorphism in sporadic and hereditary ovarian cancer risk.

This finding is in contrast to our observation that the *PHB *polymorphism acts as a modifier of *BRCA1*-associated breast cancer risk in a similar collective of 258 breast cancer patients and 258 matched controls [10]. The most likely explanation for the difference of effect between breast cancer and ovarian cancer patients within our study set is in the underlying disease mechanisms involved in breast cancer compared to ovarian cancer. At this stage what these differences are remains to be resolved. However, it also cannot be ruled out that due to the smaller size of the ovarian cancer study no association was detected. Thus, larger studies are warranted to confirm these preliminary results.

## Conclusion

In summary, the 3'UTR polymorphism in *PHB *appears not to be associated with ovarian cancer risk in women carrying of one of the three Polish *BRCA1 *founder mutations even when accounting for environmental influences that are considered important in disease risk determination. The identification of genetic ovarian cancer risk modifiers remains to be accomplished.

## Competing interests

The author(s) declare that they have no competing interests.

## Authors' contributions

AJ participated in the design of the study, carried out the molecular analyses and contributed to data acquisition and interpretation, drafting and revising the manuscript. JG, JM, BG, TH, TB were involved in data acquisition. AB performed the statistical analyses. JL contributed to conception and design and secured funding. RJS participated in conception, design, data interpretation and was involved in revising the manuscript. UH coordinated the study and participated in its design, data interpretation, drafting and revising the manuscript, and secured funding. All authors have given final approval of the version to be published.

## Pre-publication history

The pre-publication history for this paper can be accessed here:


